# Application of the deep learning for the prediction of rainfall in Southern Taiwan

**DOI:** 10.1038/s41598-019-49242-6

**Published:** 2019-09-04

**Authors:** Meng-Hua Yen, Ding-Wei Liu, Yi-Chia Hsin, Chu-En Lin, Chii-Chang Chen

**Affiliations:** 1 Department of Electronic Engineering, National Chin-Yi University of Technology, 411 Taichung, Taiwan; 2 Department of Optics and Photonics, National Central University, 320 Taoyuan, Taiwan; 3 Research Center for Environmental Changes, Academia Sinica, 115 Taipei, Taiwan; 4 Lordwin Technology Inc., 804 Kaohsiung, Taiwan

**Keywords:** Applied mathematics, Information technology

## Abstract

Precipitation is useful information for assessing vital water resources, agriculture, ecosystems and hydrology. Data-driven model predictions using deep learning algorithms are promising for these purposes. Echo state network (ESN) and Deep Echo state network (DeepESN), referred to as Reservoir Computing (RC), are effective and speedy algorithms to process a large amount of data. In this study, we used the ESN and the DeepESN algorithms to analyze the meteorological hourly data from 2002 to 2014 at the Tainan Observatory in the southern Taiwan. The results show that the correlation coefficient by using the DeepESN was better than that by using the ESN and commercial neuronal network algorithms (Back-propagation network (BPN) and support vector regression (SVR), MATLAB, The MathWorks co.), and the accuracy of predicted rainfall by using the DeepESN can be significantly improved compared with those by using ESN, the BPN and the SVR. In sum, the DeepESN is a trustworthy and good method to predict rainfall; it could be applied to global climate forecasts which need high-volume data processing.

## Introduction

Taiwan is located in the subtropical monsoon climate zone, year-round rainy, and surrounded by seas, and has highly changing terrain elevation. Taiwan often suffers major disasters caused by heavy rainfall which is due to the severe weather system, such as typhoon^1–3^. Furthermore, the terrain of Taiwan is ever-changing, slope steep and sharp, so the temporal and spatial variability of rainfall is extremely uneven. It is resulting in the fact that the water is not easy to be stored. Therefore, Taiwan is still identified as the lack of water region by the United Nations although it has abundant rainfall. In addition, rainfall is an important basis for assessing water resources, agriculture, ecosystems and hydrology. Thus, how to accurately predict rainfall has been a very crucial issue in the weather forecast community worldwide.

In forecasting rainfall, satellite imageries, ground observation stations and weather balloons were used mostly. There is also radar imaging technology, but it is not yet an extensive application due to the large image data. The existing rainfall forecasting in the Central Weather Bureau of Taiwan is based on international standards, in which data collected from weather balloons twice a day, ground meteorological stations and satellite remote-sensing is adopted. Sequentially, a temporal interval of 12 hours is set to do rainfall forecast (quantitative precipitation forecast, QPF). Many scholars have focused on improving the monitoring and forecasting of weather and developing the technology of QPF to improve the ability to forecast heavy rainfall events^2,4–7^.

In recent years, the use of artificial intelligence algorithm for rainfall forecasting has attracted considerable attention^8–14^. The mechanism of rainfall forecasting is a nonlinear system in terms of mathematics. The artificial intelligence model proposed by fuzzy theory and neural network in recent years has a very good effect on dealing with nonlinear system. For example, in the hydrological flow and rainfall forecasting, there is a good development. Nayak *et al*.^11^ used the fuzzy theory to predict the outflow of the river in the Mandala watershed in India. Reinhard *et al*.^13^ improved the forecast of weather radar with feed-forward neural networks. However, how to apply these artificial intelligence algorithms actually to rainfall forecasting of large amounts of data is another important issue. The computer systems, that can be applied to artificial intelligence operations and have capabilities of high-speed computing and large data processing, are often difficult to obtain. Therefore, photonic neural networks might be another solution to this problem, because of its ultra-high computing power^15^. Reservoir Computing (RC)^16,17^ is one specific supervised learning technique of Recurrent Neural Networks (RNNs), is a very advanced approach for processing time dependent data or information and is often used in photonic neural networks to process data^18,19^. The most important advantage of RC is that the training algorithms are efficient and converge quickly to the optimum because only output layer is trained. Photonic neural networks by using RC can quickly analyze the data and make predictions^15,20^. Even the concept for standalone physical reservoir computers by the RC has been verified^21^. For example, Larger *et al*.^22^ showed that the performances reach one million words per second, with very low word error rate. In addition to the photonic application of the RC, a previous study shows that using a novel recurrent neural network–echo state network (ESN)^23^, which is one of the most representative RC, to predict the next closing price in stock markets^24^. Furthermore, the deep echo state network (DeepESN) model, which is an enhanced ESN model, has been highly valued in recent years^25,26^. The DeepESN model opens the way for extremely efficient approach for designing deep neural networks for temporal data^25–31^. The advantages of the DeepESN approach includes the multiple temporal representations, richness of reservoir states, memory capacity and efficiency^26^. In other words, although many other methods are able to be applied in rainfall forecasting^8–13,32–35^, they might have a problem of computational efficiency in the future when dealing with a larger amount of meteorological data. Therefore, using the RC method to predict rainfall is a promising method.

The main purpose of this study is to develop a forecasting model of rainfall using RC and to investigate the possible factors in governing the rainfall forecast in the southern Taiwan. To the best of our knowledge, this is the first study that rainfall is forecasted by using RC. In summary, we present the direct evidence for the system performance and the effect of rainfall prediction.

## Results and Discussion

### Performance evaluation of forecasting model

Evaluation of the established model in this study can be divided into three stages. The 1^st^ stage is to find out the best algorithm (model) by comparing several statistical quantities, such as root mean square error (RMSE), normalized root mean squared error (NRMSE), and correlation coefficient (γ). Echo State Network (ESN) model and Deep Echo State Network (DeepESN) model are used to forecast the rainfall in the southern Taiwan. After training with the data since the beginning of 2002, we can input the test data (i.e, the remaining data) into the network in the established model to forecast rainfall and RMSE, NRMSE, and γ are further calculated from the observed precipitation and the predicted value. The 2^nd^ stage is to verify the feasibility of the best model with neural network model provided by commercial software (Neural Network Toolbox of MATLAB software) and compare the predicted results of the best model with those in the literature. In the 3^rd^ stage, we adopt some metrics items for quantitative precipitation forecasts (QPF) in consideration of rainfall greater than a certain threshold to evaluate the performance of the model forecast, such as probability of detection (POD), false alarm ratio (FAR) and threat score (TS)^36^. Note that the calculations in the 1^st^ and 2^nd^ stages are executed based on the original predicted time series, in which “negative values” are included; however, in the 3rd stage, the negative values in the predicted time series are set to “zero” with consideration of a real world (or physics), i.e., no negative rainfall appears in a real world.

In this study, the meteorological data were obtained from the two observational stations (Zengwen Observatory information and Yujing Observatory information in the Tainan City, Taiwan) and the Sea Level Center (University of Hawaii). A total of seven parameters, including air pressure, temperature, humidity, wind speed, wind direction, precipitation, and sea level. Because the meteorological data of the Zengwen Observatory information (102,500 points of data) is much larger than that of the Yujing Observatory information (12,700 points of data). Therefore, we first used the data of the Zengwen Observatory information (Obs_zen_) to carry out ESN model and DeepESN model training and preliminary verification. After completing the training of the ESN model and the DeepESN model, we replace the meteorological data of the Obs_zen_ with those of Yujing Observatory information (Obs_yuj_) to predict the rainfall around the Yujing Observatory information to examine the applicability of the trained ESN model and the DeepESN model for other location. The details of the ESN model, the DeepESN model, definitions of the RMSE, NRMSE, γ, POD, FAR, and TS, and details of data adopted will be shown in the method section below.

**Figure 1:**
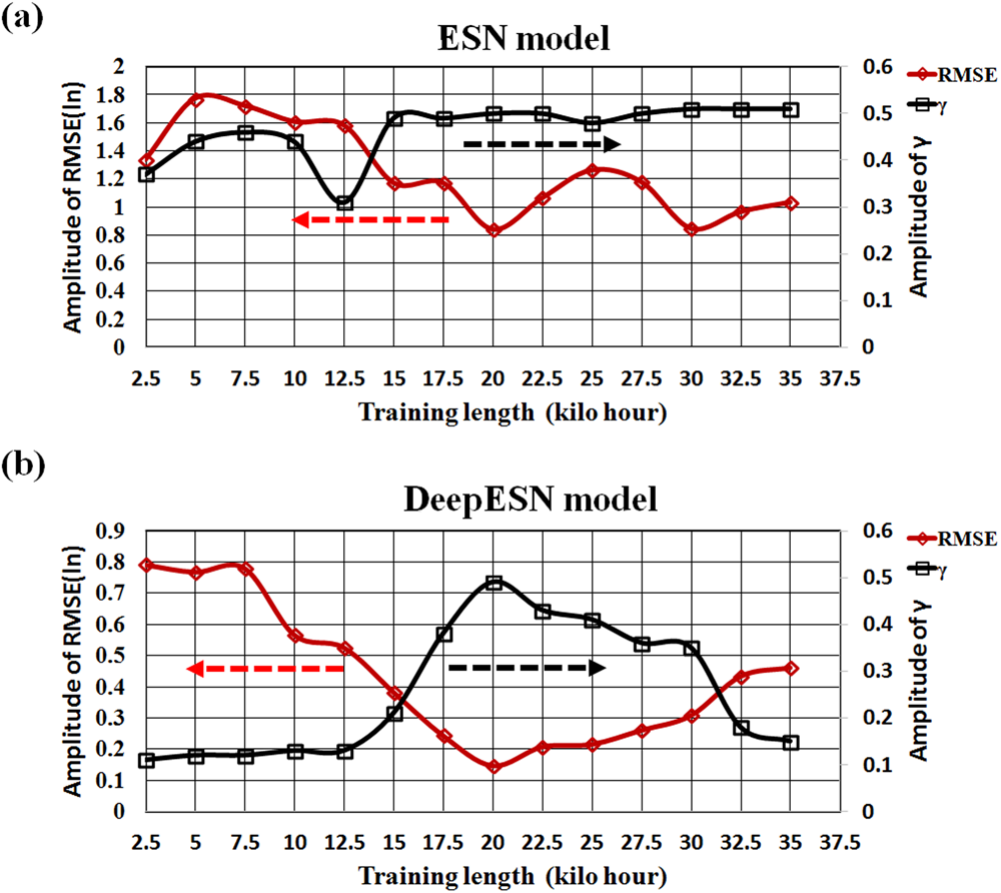
Effect of the training length for the ESN model (**a**) and DeepESN model (**b**). RMSE is scaled by natural logarithm (ln).

According to previous study^37^, the selection of training data has a very large impact on the model prediction accuracy, and the prediction accuracy is quite sensitive to the length of training data. The data from the Obs_zen_ was used for testing. First, we adjust the training length at an interval of 2,500 hours from 2,500 to 35,000 hours and calculate the RMSE and γ to find out an optimal training length in order to discuss the sensitivity of the number of training sessions to the rainfall forecast. The results are depicted in Fig. 1 for the ESN model and DeepESN model. When the training length exceeds 15,000 hours, the γ value for the ESN model tends to be stable (Fig. 1a). As the training length increases, the RMSE for the ESN model is significantly reduced and bottoms out at 20,000 hours with a minimum of 6.95; afterwards, it fluctuates between 6.95 and 18.3. This outcome indicates that the prediction skill of ESN model stabilizes when the training length is longer than 20,000 hours, i.e., the length of training data beyond 20,000 hours can no longer improve the prediction accuracy in the model. For the DeepESN model prediction (Fig. 1b), the RMSE and γ values significantly change after the training lengths of 7500 hours and 12,500 hours are selected, respectively. Similar to the ESN model, both RMSE and γ can get the best value until the training length of 20,000 hours is adopted, i.e., the RMSE andγvalues start to deteriorate again after this point. Therefore, the optimal training length of 20,000 hours is utilized to establish the best ESN/DeepESN model for the further analyses.

**Figure 2:**
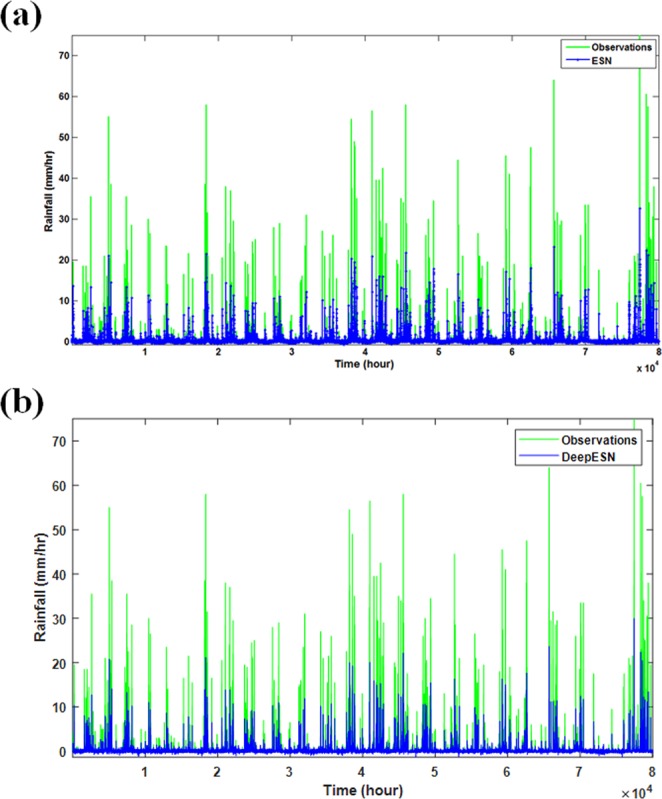
Comparison of observed rainfall (green curve) at the Zengwen Observatory information in the Tainan City with the predicted rainfall (blue curve) by using ESN model (**a**) and DeepESN model (**b**).

Excluding optimized training length of 20,000 hours from the data of the Obs_zen_, the remaining data length of 82,500 hours will be adopted to predict rainfall in the southern Taiwan and used to examine the performances of the ESN/DeepESN model. Figure 2 compares the time series of predicted rainfall with the observed rainfall in the corresponding period for the ESN model and DeepESN model. Visually, there is no significant difference between predicted rainfall in the ESN model and the DeepESN model. To further evaluate the model performances for the ESN model and DeepESN model, the RMSE, NRMSE and γ are firstly calculated to be the judgment indices. The precipitation predicted by the trained ESN model (Number 1 in Table 1) gives the RMSE, NRMSE and γ as 6.95, 0.093 and 0.494, respectively, while the RMSE, NRMSE and γ for the DeepESN (Number 2 in Table 1) are 1.51, 0.02 and 0.507, respectively.

**Table 1:** Comparison of different modeling methods for the rainfall prediction. The data from the Obs_Zen_ was used for the number 1(ESN) and 2(DeepESN), 3(BPN), 4(SVR). The data from the Obs_yuj_ was only used for the number 1(ESN) and 2(DeepESN).

Model		RMSE	NRMSE	γ	POD	FAR	TS	Time interval of data (hour)
1. ESN	Obs_Zen_	6.95	0.093	0.457	0.98	0.02	0.96	1
	Obs_yuj_	7.15	0.083	0.457	0.97	0.03	0.95	1
2. DeepESN	Obs_Zen_	1.51	0.02	0.507	0.98	0.02	0.96	1
	Obs_yuj_	2.08	0.018	0.457	0.97	0.03	0.95	1
3. BPN (MATLAB)	Trainlm	2.11	0.02	0.31	0.98	0.02	0.96	1
	trainbfg	2.12	0.028	0.3	0.97	0.03	0.96	1
4. SVR (MATLAB)		1.66	0.026	0.28	0.98	0.02	0.97	1
5. ECMWF^25^		7.15	N/A	0.7	0.8	0.2	N/A	24

Here the Obs_Zen_ is the data from Zengwen Observatory, the Obs_yuj_ is the data from Yujing Observatory, the ECMWF is European Centre for Medium-Range Weather Forecasts.

Besides, the meteorological data from the Obs_yuj_ were further adopted as the input for the ESN/DeepESN model testing to examine whether the ESN/DeepESN model trained by the Obs_zen_ can be directly applied to make rainfall prediction at other observatories. For the ESN model, the RMSE, NRMSE and γ are 7.15, 0.083 and 0.457, respectively (Number 1 in Table 1). By comparing the statistical quantities between the two stations (Obs_zen_ and Obs_yuj_), all quantities are quite close. Furthermore, the RMSE, NRMSE and γ for DeepESN model are 2.08, 0.018 and 0.457, respectively (Number 2 in Table 1). Similarly, difference of the evaluation items between the Obszen and Obsyuj is insignificant (as shown in Table 1). In sum, the present ESN/DeepESN model is convincible enough to make prediction of rainfall in the southern Taiwan.

Different from previous researches on artificial intelligence is that the present ESN/DeepESN model is computationally efficient and does not require the use of high-speed and expensive computing equipment with a high speed Graphics Processing Unit (GPU). In this study, standard personal computer is utilized to perform the prediction and configures the Central Processing Unit(CPU) of Intel Core i5 (2.20 GHz), operating system of Microsoft Windows 10 Professional x64, memory of 8 GB RAM, and a graphics card with a normal module (Nvidia, Geforce 830M).

By comparing the results based on DeepESN model with the ESN model, we found that the evaluations items are able to be improved by the DeepESN with a slight increase of computing time. For example: the RMSE value is reduced from 6.95 to 1.5 (about 4.7 times lower). This improves not only the accuracy of the prediction method, but also the correctness of the rainfall prediction. In addition, the number of negative values in the predicted time series (80,000) is about 28,000. Although the number is about 1/3, most of the negative values are small and the average is −0.12 with the extreme of −1.32). In spite of this, the statistical comparison still evidences that the prediction skill based on the best model is better than others. Therefore, we believe that this method is feasible for rainfall prediction.

In the second stage, we compare the predicted rainfall with the neural network model provided by commercial software (Neural Network Toolbox of MATLAB software) and rainfall prediction skill reported in the literature (Number 3–5 in Table 1) in order to further verify the feasibility of the ESN/DeepESN model. It is pointed out that the Back-Propagation Neural Network (BPN) and Support Vector Regression (SVR) are two kinds of neural network models which have been extensively applied to rainfall forecasting^12,14,38–41^. The architecture of BPN and SVR will be shown in the following method sections. In the BPN model, we use two different training functions (trainlm and trainbfg) provided by MATLAB to execute the training and rainfall prediction based on the same hourly meteorological data at the Obs_zen_ and standardization procedure adopted for the ESN/DeepESN model. In the SVR model, we use one training function provided by MATLAB to execute the same procedures. By comparing the ESN/DeepESN model with the BPN and SVR models (Number 1–4 in Table 1). The correlation coefficients for the ESN/DeepESN are significant greater than those for the BNP and SVR model, while the ESN model produces much greater RMSE and NRMSE than the other three models. In addition, the ESN/DeepESN (3 min for the 20,000 hours training and the 80,000 hours prediction) has shorter calculation time than the BPN (6 min) and the SVR (10 min). Therefore, the DeepESN shows the better performance than the BPN and the SVR. These facts indicate that the DeepESN, on the whole, has the best performance than the others three models.

Number 5 in Table 1 shows the prediction skill of rainfall reported in the literature. Although the region, the forecast model and the time period adopted to perform the forecast are different from the present study, it can still be used as a reference to quantitatively compare the performance of the model established in the present study. The values of the POD and FAR from the ESN/DeepESN, the BPN and the SVR (Number 1–4 in Table 1) are obviously better than those from the ECMWF. In brief, the DeepESN method used in this study is a reliable method to predict rainfall according to the above comparisons.

In the third stage, by considering QPF as a forecast of rain greater than a certain threshold, we further compare the POD, FAR, and TS in a 2 × 2 contingency table (see method section for details) with the threshold of 2.5 mm/hr, which is based on the rainfall classification method from American Meteorological Society^42^. Before calculating the metrics items of the rainfall forecast listed in Table 1, the predicted negative values of the rainfall forecast are adjusted to zero, which is in line with the reality, i.e., no negative rainfall can be found in the real world. Result shows that the precipitation predicted by the trained DeepESN model gives the POD above 0.97, the FAR below 0.03, and the TS above 0.95 (Number 2 in Table 1). Comparing the results by using the DeepESN with those by the other models (Number 1, 3 and 4 in Table 1), similar outcomes are obtained, indicating that the DeepESN is a reliable way to predict rainfall.

Finally, in order to find out the dominant factors controlling the rainfall prediction, we discuss the effect of the parameters of each input. There are several methods for reducing the input parameters for the artificial neural network, such as principal component analysis (PCA) and Autoencoder. We performed the PCA method, in anticipation of finding the most important parameters. The results show that most important parameters are the rainfall, the pressure and the humidity. In addition, we use a method of the parameter adjustment (i.e., alternatively taking an input parameter off) to adjust the contents of the input data (testing data) from the Obs_zen_ obtained by the pretreatment based on the DeepESN model. With turning off a parameter each time by adjusting the parameter to the extreme value (i.e., +1 in this study) of the mapminmax standardization, as the manipulation of the cause, the remaining six parameters as a control change. The results are shown in Table 2. The definition of changing rate of the correlation coefficient is described in method section below. Therefore, if the parameter has the greater effect on the rainfall forecast, the larger change shows up. It is expected that the performance of the DeepESN model reduces dramatically when the rainfall is not taken into account in the prediction, indicating that rainfall itself is the most important parameter for the rainfall forecast in the DeepESN model. The second and third governing factors are the pressure and humidity, respectively. In sum, both the PCA method and our method represent the key parameters of rainfall, air pressure and humidity for rainfall forecast. Therefore it might be possible to move towards the weight/specific gravity adjustment of these parameters to improve the model in the future.

**Table 2:** The effect of each input parameter on the DeepESN model was evaluated by the value of γ(% change).

	γ	Amount of change (%)	RMSE
Original	0.507	N/A	1.51
Pressure	0.355	−30.05	1.8
Temperature	0.489	−3.63	1.56
Humidity	0.463	−8.70	1.57
Wind speed	0.487	−3.86	1.55
Wind direction	0.499	−1.60	1.52
Sea level	0.501	−1.16	1.52
Rainfall	0.317	−37.44	1.69

Based on the above results, we reduce the number of input parameters in the ESN/DeepESN model from seven to three, which include rainfall, pressure and humidity. Then, we repeat the whole procedures which include the data training and data testing. In the case, the RMSE, NRMSE, and γ are 1.51, 0.02 and 0.518, respectively. This outcome indicates that removing the irrelevant parameters can improve the performance of rainfall prediction. In this study, although a small number of parameters can improve performance of prediction, it might be a special case. For the deep learning, more input parameters might get better performance for model training and testing. In the future, we will collect more meteorological data from other observatory in the southern region of Taiwan to execute the rainfall forecasting, and improve the accuracy of rainfall forecast.

## Conclusion

In this study, we demonstrate that rainfall prediction can be achieved by means of the neural networks (the ESN/DeepESN model). Through the actual test and verification, it is proved that rainfall forecasting can be performed by using the ESN/DeepESN model. The performance of DeepESN model is better than those of the ESN and commercial neuronal network algorithms (Back-propagation network and Supporting Vector Regression, MATLAB, The MathWorks co.). Therefore, the DeepESN is a better model to predict rainfall than the other models. Finally, we examine the effect of each input parameter by taking an input parameter off alternatively based on the DeepESN model. It shows that the rainfall, the pressure and the humidity are the most crucial parameters, and highly influence the performances of rainfall prediction in the Dee1pESN model. In conclusion, the DeepESN is a reliable tool to predict rainfall.

## Data and Methods

### Observations

In this study, the data was obtained from the Central Weather Bureau of Taiwan (https://www.cwb.gov.tw/V7/index.htm) and the Sea Level Center, University of Hawaii (https://uhslc.soest.hawaii.edu/). There are a total of seven parameters, including air pressure, temperature, humidity, wind speed, wind direction, precipitation, and sea level. The first six meteorological parameters are measured at Zengwen Observatory information in the Tainan City (120.497E, 23.219N), Taiwan, and the sea level is observed at the Kaohsiung Tidal Station in the Kaohsiung city (120.283E, 22.617N), Taiwan. A total of 102,500 hours of data during the period of 2002–2014 is adopted for the construction of the experimental model. In addition, meteorological data at another meteorological observatory (Yujing Observatory; 120.461°E, 23.126°N) in Tainan City, Taiwan, are used to examine the applicability of the trained ESN model for other observatories. A total of 12,700 hours of data during the period of 2013–2014 is adopted for the construction of the experimental model.

### Data preprocessing

In general, data is pre-processed in deep learning, and normalization is the most commonly used approach. Different parameters adopted in deep learning have different units of measurement and accuracy, which will affect the results of data analysis. In order to eliminate the influence of the measurement units and accuracy among these parameters, it is necessary to standardize them in the same order of magnitude and increase the comparability among the parameters. Besides, the normalization can effectively speed up the training speed of the model.

Since the values of the meteorological parameters used in this study are more concentrated, the normalization method used in this study is standardized for mapminmax. The “mapminmax” normalization results in a linear change of the original data. The conversion formula is as follows:

1 $$O=\frac{({O}_{max}-{O}_{min})\times (I-{I}_{min})}{({I}_{max}-{I}_{min})}+{O}_{min}$$

where *O*_*max*_ and *O*_*min*_ are the maximum and minimum in the output data after normalization, respectively, and *I*_*max*_ and *I*_*min*_ are the maximum and minimum in the input data, respectively. Then each dimension of the data is reoriented to a range of −1 to 1. That is, the *O*_*max*_ and *O*_*min*_ are 1 and −1, respectively.

### Model setup and predicting procedures

**Figure 3:**
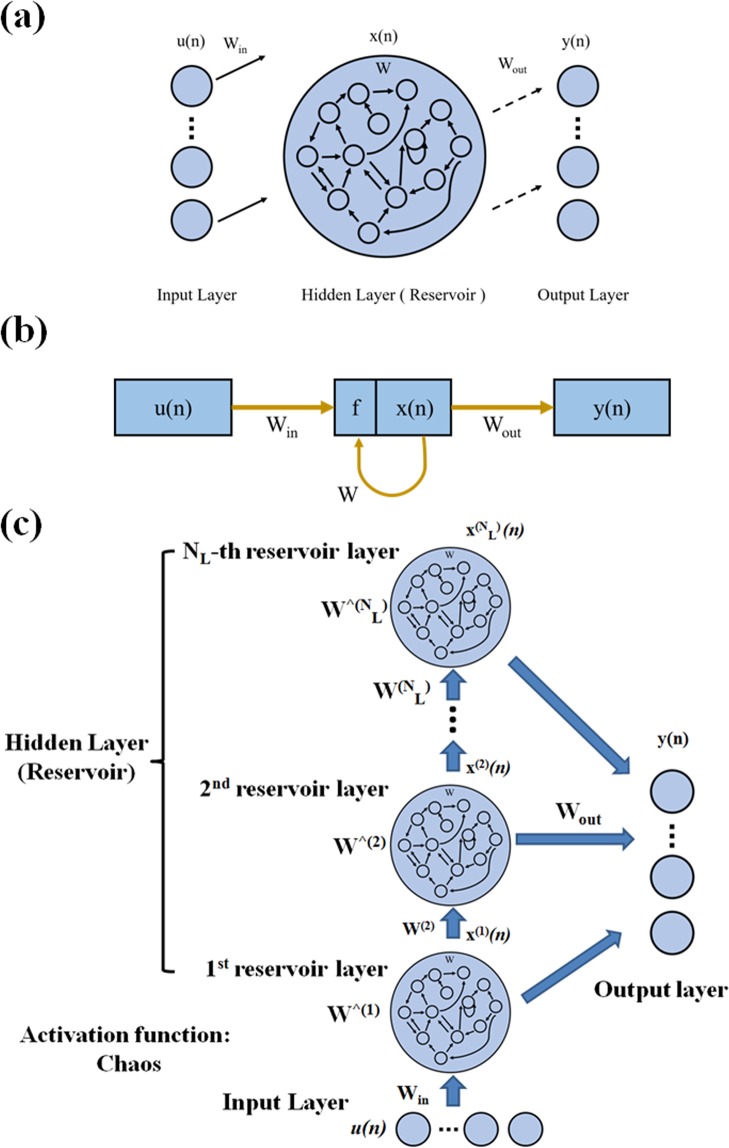
(**a**) The basic structure of ESN. It can be decomposed into three parts: input layer, hidden layer (Reservoir) and output layer. (**b**)An illustration of neural network for label processing in this study. (**c**) An architecture of a DeepESN.

Echo State Network (ESN) is one of the most representative Reservoir Computing (RC). The ESN primarily provides architecture and supervised learning principles for RNNs^23^. In general, ESN models a large number of hidden layers as its Reservoir (approximately 50 to 1000 neurons, while other techniques typically use 5 to 30 neurons). Compared to the traditional gradient-descent-based RNN training methods adapt all connection weights, including input-to-RNN, RNN-internal, and RNN-to-output weights. In ESN, only the RNN-to-output weights are adapted^16,17^. It can be seen that ESN can be trained using many linear regression algorithms to improve the performance of learning. The structure of ESN can be decomposed into three parts: an input layer, a hidden layer (reservoir) and an output layer (as shown in Fig. 3a). The network architecture is shown in Fig. 3b, in which u(n) is the input data of the network where n is the discrete time, W_in_ is untrained weight layers, f is non-linear functions (chaos), x(n) is the state vector of reservoir at time step n, W_out_ is the weights obtained after training, W is the weight matrix inside the reservoir, and y(n) is the output of the network. The activation function (f) used in ESN model was chaos. The neuron number of hidden layers as the Reservoir of the ESN model in this study is 100. Furthermore, the DeepESN is based on the ESN architecture and is enhanced. The architecture is shown in Fig. 3c. For convenience of explanation, the state transition function of the first layer of the DeepESN is defined as follows:

2 $${x}^{1}(n)=(1-{a}^{(1)}){x}^{(1)}(n-1)+{a}^{(1)}chaos({W}_{in}u(n)+{W}^{\wedge (1)}{x}^{(1)}(n-1)),$$

while for every layer *l* > 1 it is described by^26^:

3 $${x}^{(l)}(n)=(1-{a}^{(l)}){x}^{(l)}(n-1)+{a}^{(l)}chaos({W}^{(l)}{x}^{(l-1)}(n)+{W}^{\wedge (l)}{x}^{(l)}(n-1))$$

where *W*_*in*_ is the input weight matrix, *W*^*^(l)*^ is the matrix of the recurrent weights for layer *l*, *W*^*(l)*^ is the matrix relative to the inter-layer connection weights from layer *l* − 1 to layer l, *a(l)* is the leaky parameter at layer *l* and chaos represents the element-wise application of the chaos^26^.

According to previous research^26^, the network structure of the DeepESN is about 7 layers and can get good results. Therefore, architecture of the DeepESN used in this study is 7-layer architecture. The neuron number of hidden layers as the Reservoir of the DeepESN model in this study is about 700. The more detail relevant descriptions about the ESN, DeepESN or RC basic model can be found in some literatures^16,17,23,25–27,29,43–45^.

The ESN/DeepESN is used as the basic structures to process the seven meteorological and hydrological parameters, namely, pressure, temperature, humidity, wind speed, wind direction, sea level and rainfall, on a total of 102,500 time points from the Obs_zen_. The whole time series of the seven parameters are preprocessed with the mapminmax normalization. The data of the first 20,000 time points from the Obszen were utilized as the input for the ESN/DeepESN training and the remaining 82,500 data from the Obs_zen_ are adopted as the input for the ESN/DeepESN testing. The next hour of rainfall of input data is assumed as the output answer to compare with the predicted rainfall. Finally, the whole 12,700 data from the Obs_yuj_ are adopted as the input for the ESN/DeepESN testing. This can further verify the performance of the ESN/DeepESN model.

The rainfall forecast results, which are from the test data through the experimental model, were used to examine the performance of forecast. The judgment index includes three statistics: root mean square error (RMSE), normalized root mean squared error (NRMSE), and correlation coefficient(γ). The formulas are described below.

4 $${\rm{RMSE}}=\,\sqrt{\frac{{\sum }_{i=1}^{N}{(Rai{n}_{obs}-Rai{n}_{mod})}^{2}}{N}},$$

where *Rain*_*obs*_ is the rainfall from the observatory, *Rain*_*mod*_ is the predicted rainfall, and *N* is sample number.

5 $${\rm{NRMSE}}=\,\frac{RMSE}{{y}_{max}-{y}_{min}},$$

where *y*_*max*_ and *y*_*min*_ are the maximum value and minimum value, respectively, in the testing data.

6 $${\rm{\gamma }}=\frac{{\sum }_{i=1}^{N}(Rai{n}_{mod}-\overline{Rai{n}_{mod}})(Rai{n}_{obs}-\overline{Rai{n}_{obs}})}{\sqrt{{\sum }_{i=1}^{N}{(Rai{n}_{mod}-\overline{Rai{n}_{mod}})}^{2}}\sqrt{{\sum }_{i=1}^{N}{(Rai{n}_{obs}-\overline{Rai{n}_{obs}})}^{2}}},$$

**Figure 4:**
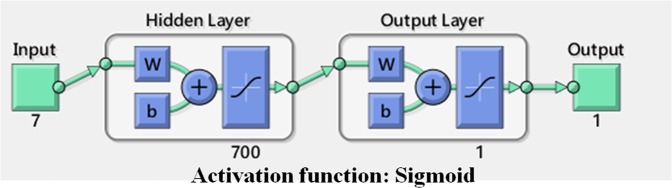
The architecture of BPN used in this study. It includes an input layer, an output layer and one hidden layer, which include 700 neuron network nodes.

In order to further verify the feasibility of the ESN/DeepESN model, we use the BPN model and the SVR model to execute rainfall prediction. The architecture of BPN includes an input layer, an output layer and one hidden layer, which include 700 neurons (Fig. 4). The training functions are trainlm and trainbfg. The activation functions of two BPN are both tansig, which is sigmoid function. In addition, we use regression toolbox in the MATLAB software to perform SVR method to predict rainfall and further compare it with the ESN/DeepESN method. The SVR method in the MATKAB toolbox is cubic SVM model. The kernel function is cubic.

**Figure 5:**
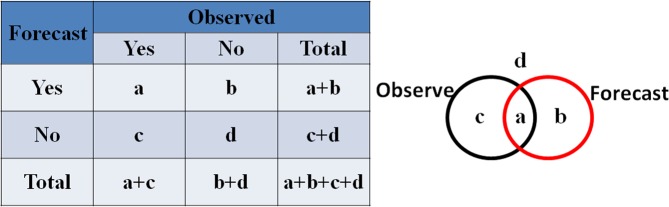
The contingency table for dichotomous forecasts of dichotomous events. An illustration on the right shows the meaning of the different parameters. POD (probability of detection) = a/(a + c), FAR (false alarm ratio) = b/(a + b) and TS (threat score) = a/(a + b + c).

In addition, if we consider quantitative precipitation forecast (QPF) as a forecast of rain greater than a certain threshold, the QPF problem can be divided into a series of 2 × 2 contingency tables, each for a different threshold value (see Fig. 5)^46,47^. Some metrics items for the QPF, such as probability of detection (POD), false alarm ratio (FAR) and threat score (TS), are able to be defined based on the numbers in the 2 × 2 contingency tables^48^. POD = a/(a + c) shows the ability to actually perceive an event when it takes place. The percentage of mistakes made when events are perceived as intense is given by the FAR, which is equal to b/(a + b). And, the threat score, TS, is defined as a/(a + b + c), which measures the ability to perceive the rare event alone by ignoring the “d counts”. The illustration on the right-hand side in Fig. 5 shows the meaning of the different parameters.

In addition, in Table 2, the changing rate of the correlation coefficient is as follows:

7 $${\rm{Amount}}\,{\rm{of}}\,{\rm{change}}=\frac{({{\rm{\gamma }}}_{c}-{{\rm{\gamma }}}_{0})}{{{\rm{\gamma }}}_{0}}\times 100 \% ,$$

where γ_c_ is the new correlation coefficient after changing the parameter and γ_o_ is the original correlation coefficient.

By comparing the results of RMSE, NRMSE, γ, POD, FAR, TS and Amount of change, the performance of the proposed network architecture in this study can be evaluated.
